# Identifying Emotions on the Basis of Neural Activation

**DOI:** 10.1371/journal.pone.0066032

**Published:** 2013-06-19

**Authors:** Karim S. Kassam, Amanda R. Markey, Vladimir L. Cherkassky, George Loewenstein, Marcel Adam Just

**Affiliations:** 1 Department of Social and Decision Sciences, Carnegie Mellon University, Pittsburgh, Pennsylvania, United States of America; 2 Department of Psychology, Carnegie Mellon University, Pittsburgh, Pennsylvania, United States of America; The University of Queensland, Australia

## Abstract

We attempt to determine the discriminability and organization of neural activation corresponding to the experience of specific emotions. Method actors were asked to self-induce nine emotional states (anger, disgust, envy, fear, happiness, lust, pride, sadness, and shame) while in an fMRI scanner. Using a Gaussian Naïve Bayes pooled variance classifier, we demonstrate the ability to identify specific emotions experienced by an individual at well over chance accuracy on the basis of: 1) neural activation of the same individual in other trials, 2) neural activation of other individuals who experienced similar trials, and 3) neural activation of the same individual to a qualitatively different type of emotion induction. Factor analysis identified valence, arousal, sociality, and lust as dimensions underlying the activation patterns. These results suggest a structure for neural representations of emotion and inform theories of emotional processing.

## Introduction

Emotional experience pervades every aspect of mental life. Emotions have profound influences on the content and style of thought [1], affecting not only our decisions and actions [2] [3] [4], but also our memories and perceptions [5] [6] [7]. Though researchers agree on the importance of emotion, there is little consensus as to their neural structure or the processes that give rise to them [8] [9] [10].

Whereas some have suggested that specific emotions such as anger and fear are universal programs evolved to deal with recurrent problems faced by our ancestors [11], others believe that emotions are socially or psychologically constructed phenomena, dependent on learning and high-level cognitive processes rather than biologically given (for a review, see [12]). The former, a biologically basic emotion view, implies a species-specific computational architecture that mediates emotional response. As such, the lack of identifiable neural signatures of emotion has represented a substantial stumbling block for this perspective. Indeed, it would be difficult to consider an emotion such as anger to be a biologically determined category if different instantiations of anger had little in common at the neural level [12]. One of the goals of the present experiment was to examine whether patterns of brain activity characteristic of specific emotions exist, and whether these patterns are to some extent common across individuals.

### The Search for Neural Correlates of Emotion

To date, more than two hundred papers have examined the neural correlates of emotional experience using fMRI and PET alone [13]. Meta-analyses of those studies have concluded that, while some regions are more active than others when participants experience certain specific emotions, no region is both consistently and specifically activated by a single emotion category [14] [13] [15] [16] [17] [18]. That is, there is little to no evidence of the existence of an anger (or sadness, disgust, happiness, etc.) module. The search for neural correlates of emotion may have been hampered, however, by outdated localization models [19], as well as the use of statistical methods not well suited to the task of identifying spatially-distributed activation signatures [20]. While the existence of a localized anger ‘module’ is unlikely, there may well exist a neural signature of anger, manifested as a distributed pattern of activity.

Rather than search for contiguous neural structures associated with specific emotions, we applied multi-voxel pattern analysis techniques to identify distributed patterns of activity associated with specific emotions [21] [22]. Such techniques allow for the possibility that neural responses to emotional stimulation occur in many brain areas simultaneously. These algorithms frequently result in increased predictive power, and recent research suggests that they hold promise for classifying emotion using neurological and physiological data [23].

In particular, multi-voxel pattern analysis (MVPA) has shown great promise in classifying the emotional content of facial, bodily, and vocal expressions. Patterns of activity in voice-sensitive cortices can be used to distinguish between angry, sad, relieved, joyful, and neutral vocal expressions [24], and these patterns generalize across speakers. Similarly, distributed patterns of activity in areas implicated in the processing of facial expressions (i.e. anterior and posterior superior temporal sulcus (STS) and frontal operculum) can be used to distinguish between facial expressions of seven emotions [25]. Recent research further shows that some of these patterns generalize across stimulus types, suggesting that they are identifying emotions *per se* rather than specific stimulus-response patterns. Patterns of activity in the medial prefrontal cortex (MPFC) as well as the STS appear to encode the emotional content of a social cue irrespective of whether that content comes from vocal, facial or bodily expression [26].

These results suggest that emotion-specific neural signatures may exist, but are open to alternative explanations. Successful categorization of social cues may derive from processing related to mental state attribution rather than emotion. A person displaying an angry face is likely to think and behave differently than one displaying a sad face, and different attributions made by participants exposed to such faces, rather than differences in felt emotion, may account for the observed patterns of neural activation [26]. Perceiving an emotion and experiencing an emotion are not one and the same. Patterns of activation relating to emotion perception have been discovered, but these patterns may relate to emotional processing, mental state attribution, or some combination of the two.

MVPA has also been used to classify emotional processes in contexts in which mental state attribution does not represent an alternative explanation [27]. Baucom and colleagues [27] presented participants with four types of images taken from the international affective picture system (IAPS [28]): high arousal negative, low arousal negative, high arousal positive, low arousal positive. Their logistic regression classifier was able to identify stimulus type from these four categories on the basis of neural activation at well over chance rates, even when trained on separate subjects (i.e., in a between-subject classification).

The present experiment builds on these previous findings. By examining a broad swath of emotional experiences (anger, disgust, envy, fear, happiness, lust, pride, sadness, and shame), we aimed to establish whether specific emotion categories have neural signatures that are both identifiable within participants and common across participants.

### Factors Underlying Emotion Representation

A second goal of the present experiment was to examine neural factors underlying successful classification of emotional states. More specifically, we sought to decompose neural activation signatures into factors that could represent core neural components of emotion representation. Each factor was expected to be manifested by a spatially-distributed pattern of activation over the set of emotions.

Several candidate dimensions emerge from the literature. Researchers have frequently identified valence and arousal as two dimensions on which the neural activation deriving from emotional stimuli can be differentiated [27] [29] [30], and these two dimensions represent the most commonly identified in dimensional theories of the psychology of emotion [31] [32] [33]. Indeed, in following up on their MVPA, Baucom and colleagues [27] found the internal representation of affect underlying their data to be based on valence and arousal.

Valence and arousal may thus represent important dimensions upon which the brain differentiates affective experiences. However, additional dimensions may also exist and emerge only when specific emotion categories that differ in ways not fully captured by valence and arousal are examined. Among the most prominent among these are approach/avoidance, and sociality (i.e. whether other people are targets of a given emotion).

Though approach/avoidance motivations correlate with valence (we approach stimuli we like and avoid those we dislike), researchers have made the case that they represent a separate dimension [34]. Angered individuals, for example, are generally motivated to approach the source of their anger (e.g. to fight), despite the stimulus’ negative valence. Approach/avoidance motivational states have frequently been linked to asymmetries in left/right frontal cortical activation, especially using EEG [17], though meta-analyses of fMRI data have failed to find consistent localizations [16].

Another factor suggested by neuroimaging data is sociality [35]. Emotions play a prominent role in guiding social interaction [36] [37]. Many stimuli frequently used to elicit emotions are inherently social (e.g. facial expressions), and a number of neural regions have been implicated in the processing of social cues specifically. Researchers frequently distinguish between emotions that are inherently social (e.g. envy) and those that are not necessarily social (e.g. disgust [38] [39]). Moreover, prior research has found that neural networks distinguish between social and non-social emotions [35].

By examining a wide range of emotional experiences, we sought to discover which of these factors (valence, arousal, approach/avoidance, sociality) jointly constitute the neural signatures of emotion.

### Experiment Overview

We applied a Gaussian Naïve Bayes pooled variance classifier [40] to neuroimaging data to classify a broad variety of emotional experiences. Participants were method actors experienced with entering and exiting emotional states on cue. Prior to the neuroimaging session, each wrote scenarios that had made them feel or would make them feel emotional states denoted by 18 words grouped into nine emotion categories: anger(angry, enraged), disgust(disgusted, revulsed), envy(envious, jealous), fear(afraid, frightened), happiness(happy, joyous), lust(lustful, horny), pride(proud, admirable), sadness(sad, gloomy), and shame(ashamed, embarrassed). Participants also wrote a calm scenario that was used as a baseline. Before entering the scanner, participants engaged in a practice session designed to familiarize them with the experimental task. In the scanner, participants were presented with each word six times in random order. They were given nine seconds to imagine the scenario and enter the appropriate emotional state, followed by eleven seconds to exit that state, rate their emotional intensity, and prepare for the next trial. Once this portion of the session was complete, participants viewed 12 disgusting images and 12 calm/neutral images in random order. We examined the classifier’s ability to identify the specific emotion experienced within-individuals, between-individuals, and between-modalities.

## Methods

### Participants

Ten adults (eight right-handed females, two right-handed males, *M*_AGE_ = 20.7, *SD* = 2.1) from the Carnegie Mellon Drama Community participated and gave written informed consent approved by the Carnegie Mellon Institutional Review Board. Participants were recruited via flyers, email and in person solicitation from an undergraduate acting class. One additional male participant was scanned but excluded from the analysis because he received different instructions due to experimenter error.

### Task

Prior to the scan, participants wrote scenarios for each of the 18 emotion words, indicated whether the scenario was derived from a past experience or an imagined experience, and assessed the scenario on seven point scales of valence, arousal, certainty, control and attention, as well as the nine emotion categories. Each participant was free to choose any scenario for a given emotion, and could write as little or as much detail as they desired. There was no attempt to impose consistency across participants in their choice of scenarios. Participants were encouraged to review their scenarios immediately prior to the scan. During the scan, participants’ task was to actively experience emotions on cue using the scenarios they had previously constructed.

### Scanning Paradigms

Emotion words were presented in two separate scans lasting approximately 19 minutes each with a five-minute rest interval in between. The 18 emotion words were each presented six times in random order. Each word was presented for 9 s: 1 s in isolation followed by 8 s on screen with a pie chart that filled at a rate of one piece per second for eight seconds. Participants were asked to attain maximal emotional experience at the end of this 9 s period, when the pie was full. A fixation cross was then presented for 7 s, followed by a 4 s interval during which participants rated the intensity of their emotional experience on a 4-pt scale using two handheld computer mice. The word *calm* was presented for 40 s at the beginning and end of each of the two scans.

Following the second emotional word scan, participants viewed 12 disgusting and 12 neutral 450×450 pixel pictures presented in random order. The majority of pictures were taken from the IAPS picture set [28] (all images are available from the authors upon request). None of the pictures had been previously viewed by the participants. As in the emotion word paradigm, the word *calm* was presented for 40 s at the beginning and end of the 10-minute image scan. The 24 pictures were each presented once for 9 s along with a pie chart. A fixation cross was then presented for 7 s, followed by a 4 s interval during which participants rated their level of disgust on a 4-pt scale. Participants were instructed simply to view the images.

### fMRI Procedures

Functional images were acquired on a Siemens Verio (Erlangen, Germany) 3.0T scanner at the Scientific Imaging & Brain Research Center of Carnegie Mellon University using a gradient echo EPI pulse sequence with TR = 2000 ms, TE = 30 ms and a 79° flip angle. Siemens 32 channel receive only coil and parallel imaging (GRAPPA) with an acceleration factor of 2 were used. Thirty-four 3 mm thick AC-PC aligned oblique-axial slices were imaged. The acquisition matrix was 64×64, with 3 mm×3 mm×3 mm voxels.

### Data Preprocessing

Initial data processing was performed using SPM2 (Wellcome Department of Cognitive Neurology, London). The data were corrected for slice timing, motion, and linear trend, and were normalized into MNI space (3.125 mm×3.125 mm×3 mm voxels). Gray matter voxels were assigned to anatomical areas using Anatomical Automatic Labeling (AAL) masks. Where noted below, the images were partitioned into several bilateral brain areas using AAL masks: frontal, parietal, temporal, and occipital. In addition, masks corresponding to all AAL-defined subcortical areas (excluding cerebellum) and cingulate areas (consisting of anterior and posterior cingulate) were used.

The percent signal change relative to fixation was computed at each gray matter voxel for each stimulus presentation. The primary input measure for analyses consisted of the mean of three brain images acquired within a 6 s window. The window was offset 8 s from the stimulus onset (to account for the delay in hemodynamic response and to capture the highest level of emotional intensity). For the picture scan, the mean of two images acquired within a 4 s window and an offset by 4 s was used. The intensities of the voxels in this mean image for each word were normalized (mean = 0, SD = 1). The data were not spatially smoothed.

### Machine Learning Overview

The machine learning techniques used can be separated into three stages: 1) algorithmic selection of a small set of voxels believed to be useful for classification; 2) training of a classifier on a subset of the data; and 3) testing of the classifier on an independent subset of the data. The training and testing used cross-validation procedures that iterated through cycles of all possible partitions of the data into training and testing datasets. The training set and test set were always independent.

Rather than examine the performance of multiple classifiers and different numbers of parameters, we chose our classifier and parameters on the basis of optimizations performed in several previous studies [40] [41]. Where we did explore parameters, results are noted explicitly. We used a Gaussian Naïve Bayes (GNB) classifier, and chose voxels as described below. We note that while assumptions of the Gaussian Naive Bayes classifier are not fully satisfied by the typical fMRI dataset, they frequently yield good results even when those assumptions are not met [42]. In line with this demonstrated robustness, the GNB classifier appears appropriate here, as demonstrated by cross-validated estimates of the GNB classification rank accuracy. (As a check on the possible idiosyncrasies of GNB, the within-subject classification of emotions was repeated using logistic regression, resulting in the same mean rank accuracy (0.84) across participants.).

### Voxel Selection

Analyses focused on a small subset of all the voxels in the brain, i.e. those for which their *activation profile* over the 18 emotional words was most *stable* across the multiple presentations of the set of words. Only the activation levels of relatively stable voxels were assumed to provide information about emotion. A voxel’s stability was computed as the average pairwise correlation between its 18-word activation profiles across the multiple presentations that served as input for a given model (the number of presentations over which stability was computed was four or six, depending on the analysis). Here the 18-word activation profile of a voxel for a particular presentation refers to the vector of 18 responses of that voxel to the words during that presentation. A stable voxel is thus one that responds similarly to the 18-word stimulus set each time the set is presented. For the between subjects classification, we selected the voxels that were most stable across the 18 words for the nine participants in the training set, excluding the test participant. For the picture classification, all 6 presentations of the emotional words were used to compute stability.

In order to equate the total brain volume used for classification with that of previous mental state classification studies [40] [43], 240 of the approximately 35,000 voxels per participant were selected for use in classification based on their stability. Thus, our model fit parameters for 240 variance estimates and 9×240 estimates for mean voxel activation. An exploration of the effect of the number of voxels selected (in a range from 40 to 500 voxels) resulted in only slight variation in mean within-subject rank accuracy (in a range from 0.80 to 0.84). To estimate the false discovery rate in the selection of the 240 most stable voxels per subject, p-values for stability scores were computed using a Monte Carlo simulation with 100,000 iterations. For all participants, these 240 voxels survive conservative Bonferroni correction (all corrected p’s <.019).

### Classifier Training

In a second stage, a subset of the data (four of the six presentations in the within-participant classification) was used to train a classifier to associate fMRI data patterns with the set of nine emotion labels. A classifier is a mapping function *f* of the form: *f: voxel activation levels*→Y*_i_*, *i* = 1,…,9, where Y*_i_* are the 9 emotions, and where the *voxel activation levels* are the 240 mean activation levels of the selected voxels. We used a Gaussian Naïve Bayes (GNB)-pooled variance classifier, i.e. a generative classifier that models the joint distribution of class Y and attributes and assumes the attributes *X_1_,…,X_n_* (n = 240) are conditionally independent given Y. The classification rule is:
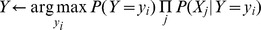
where *P(X|*Y* = y_i_)* is modeled as a Gaussian distribution whose mean and variance are estimated from the training data. In GNB-pooled variance, the variance of attribute *X_j_* is assumed to be the same for all classes. This single variance is estimated by the sample variance of the pooled data for *X_j_* taken from all classes (with the class mean subtracted from each value). We include the prior term, P(Y = yi), for the sake of completeness. The present analyses use a flat prior, as the experiment contains equal numbers of exemplars for all items.

### Classifier Testing

The classifier was tested on the mean of the two left-out presentations of each word. This procedure was reiterated for all 15 possible combinations of leaving out two presentations (following convention, we refer to such combinations as “folds” in the text below). Between-participant classification excluded data of the test participant from the training set. For picture classification, training included all presentations of the emotion words.

The *rank accuracy* of the classification performance was computed as the normalized rank of the correct label in the classifier’s posterior-probability-ordered list of classes [40]. A rank accuracy was obtained for each fold, and these rank accuracies were averaged, producing a single value characterizing the prediction accuracy for each word. Finally, the mean rank accuracy across words was computed.

### Factor Analysis

To factor the neural activity associated with the 18 different emotion words into different components shared across participants and brain regions, we used a two-level exploratory factor analysis based on principal axis factoring with varimax rotation. At the first level, a separate factor analysis was run on each participant’s data, using as input the matrix of intercorrelations among the activation profiles of the 600 stable voxels in the brain. These 600 voxels were selected from 6 bilateral brain areas (frontal lobe, temporal lobe, parietal lobe, occipital lobe, cingulate cortex, and subcortical areas excluding cerebellum), taking the 100 most stable voxels within the corresponding area. The goal of each of these first-level (within subject) factor analyses was to obtain a set of subject-specific factors describing a distributed network involved in the representation of emotion. Next, a second-level (between subject) factor analysis was run to identify factors that were common across participants. The input to the second-level analysis consisted of the scores of all first-level factors (ten dominant first-level factors were obtained from each participant; for more detail on the factor analysis methods, see [40]). We restricted analysis to the seven largest second-level factors, all with eigenvalues greater than one. Additional factors produced diminishing returns in characterizing the voxel activation profiles. Factor loading matrices from all first-level and second-level analyses were also used to create a mapping between factors and voxels. For the first-level analyses, a voxel was uniquely assigned to one of the ten first-level factors for which it had the highest (absolute value) loading, provided that this loading was above a threshold value of 0.4. Similarly, for the second level analysis, a first-level factor was uniquely assigned to one of the seven second-level factors for which it had the highest (absolute value) loading, provided that this loading has was greater than 0.4. Considered together, the above mappings allowed us to assign a set of voxels to each of the second-level factors.

To interpret the factors emerging from this data-driven method, four main sources of information were used: 1) factor scores, indicating how the 18 emotions were ranked by a given factor; 2) locations of voxels underlying each factor, and previous fMRI research showing differential activation in these locations; 3) correlation of factor scores with participant ratings of the emotions along the dimensions of valence and arousal; and 4) correlation of factor scores and ratings of the emotion words by an independent, online sample of 60 participants.

## Results

**Figure 1:**
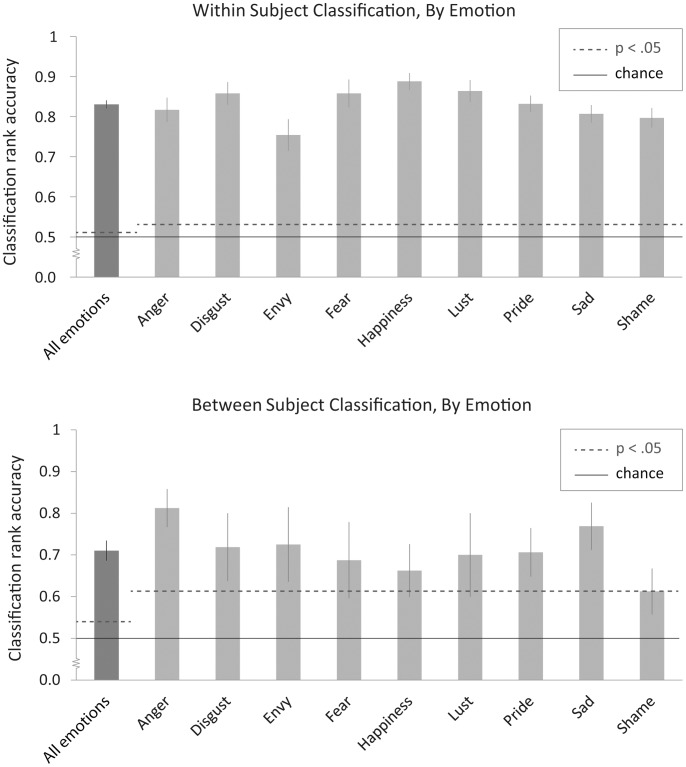
Within-subject and between-subject classification rank accuracies by emotion. Error bars represent standard error.

We first examined the ability of our classifier to identify a participant’s emotion on a particular trial on the basis of his/her neural activation during the other trials. We report the mean rank accuracy of the classification performance, that is, the percentile rank of the correct emotion category in the classifier’s posterior-probability-ordered list of emotions, averaged across the 15 ways of choosing four of six presentations. If the classification were operating at chance level, one would expect a mean rank accuracy of 0.50, indicating that the correct emotion appeared on average in the fifth position in the classifier’s ranked list of all nine emotions. Across participants, the rank accuracies for this within-subject analysis ranged from 0.72 to 0.90, with an average of 0.84, well above the chance classification rate of 0.5 (random permutation testing revealed that a mean rank accuracy greater than.51 would be significant at the p = .05 level). Mean rank accuracies for specific emotions averaged across participants ranged from 0.77 to 0.89 (all *p*’s <.05, see Figure 1). There were no significant differences between pairs of words from the same emotion category (all uncorrected *p’*s >.05). In sum, a participant’s neural activation patterns on one subset of trials could be used to reliably identify their emotions on a separate subset of held-out trials, indicating that participants exhibited consistent patterns of neural activation for all emotion categories.

Next we examined whether a participant’s specific emotions could be identified on the basis of other participants’ activation patterns. For these tests, the emotions experienced by each participant were identified using a classifier trained on the activation data from the other nine participants. Despite the challenges presented by individual variability in functional organization and methodological difficulties in normalizing morphological differences [27], the classifier achieved a mean rank accuracy of 0.70, well above chance levels (rank accuracy of 0.56 significant at the p = .01 level), with individual accuracies ranging from 0.51 to 0.81. Mean rank accuracies for specific emotions ranged from 0.61 to 0.81 (all *p*’s <.05, see Figure 1). Our classifier predicted the emotions experienced using activation patterns of other participants at significantly better than chance levels for eight of ten participants (see Table 1), suggesting that the neural correlates of emotional experience share significant commonality across individuals.

**Table 1:** Classification Accuracy for each Subject.

Subject	Within Subject	Between Subject	Disgust Picture Classification
1	0.87	0.77	1.00
2	0.82	0.60	1.00
3	0.72	0.51	0.75
4	0.85	0.65	1.00
5	0.90	0.79	1.00
6	0.81	0.80	0.88
7	0.81	0.80	0.88
8	0.87	0.81	1.00
9	0.88	0.72	0.63
10	0.84	0.67	1.00
**Mean**	**0.84**	**0.71**	**0.91**

Finally, we investigated whether patterns of activation observed in self-induced emotion trials could predict the emotional content of a stimulus from an entirely different modality. We trained a classifier using participants’ neural activation during word-cued self-induced emotions, and tested whether it could identify the emotional content of a visual image. Successful classification would indicate that the activations observed correspond to emotional experience in general, rather than to self-induced remembered or imagined emotional experiences specifically. This classification identified responses to disgust pictures with a rank accuracy of 0.91, well above chance rates (rank accuracy of 0.74 significant at the *p* = .01 level). With nine emotions to choose from, the classifier listed disgust as the most likely emotion 60% of the time, and as one of its top two guesses 80% of the time. Thus, even though the classifier had not encountered neural activation in response to pictures, it was able to accurately identify the emotional content of pictures. In contrast, the percentile rank of disgust classification for neutral pictures was 0.60 (there was no “neutral” category in the training set), significantly lower than the classification rate for disgust pictures (paired *t*(9) = 3.48, *p*<.01). The results demonstrate a consistency in the neural representation to qualitatively different stimuli for at least one specific emotion.

**Figure 2:**
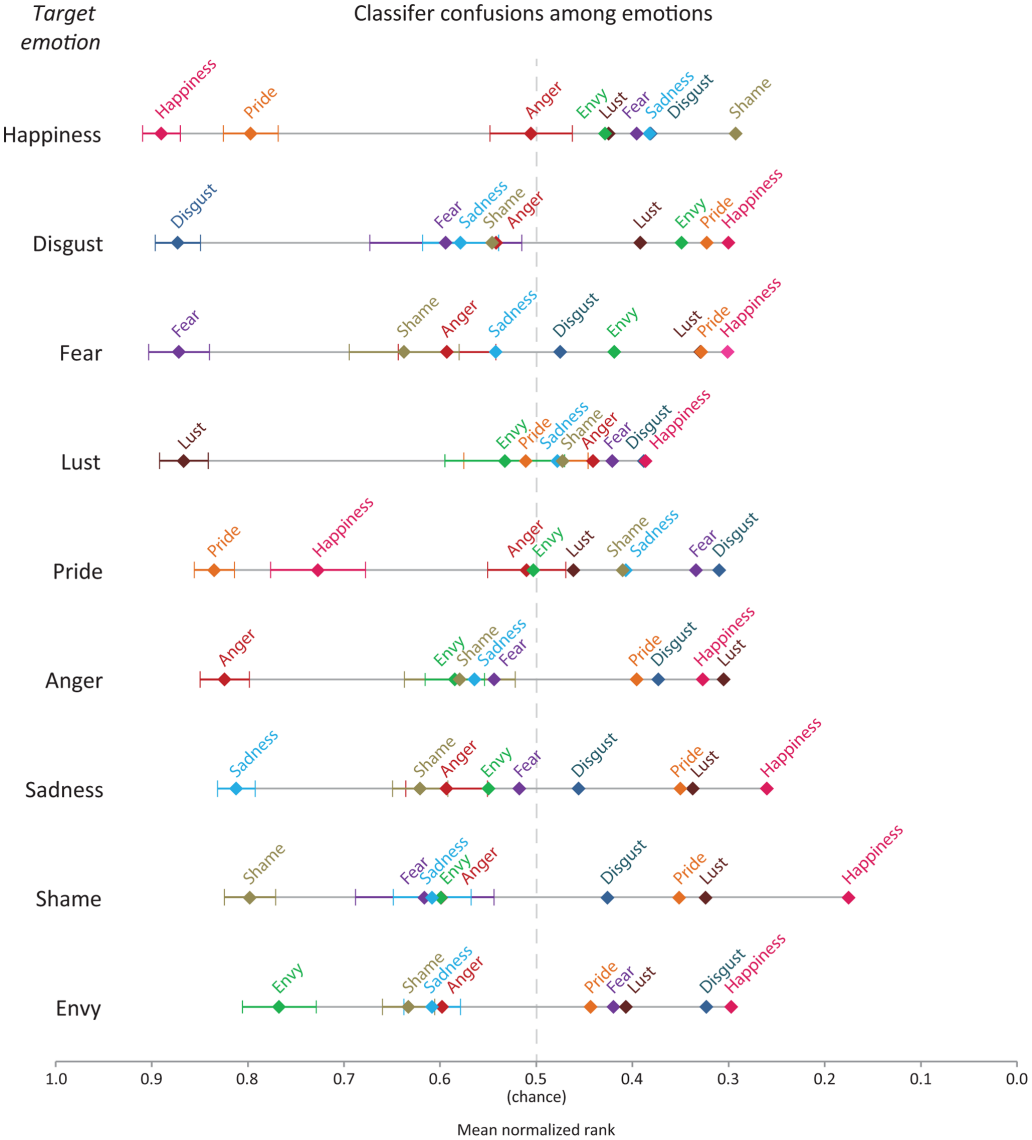
Average normalized ranks for all emotions derived from within-subject identification. Each line illustrates, for the target emotion listed on the left, the average normalized rank of classifier guesses. Error bars shown for the three highest ranked emotions represent standard error. Standard errors for all emotions ranged from 0.02 to 0.10, with a mean standard error for average positions of 0.06. See text for additional details.

The average normalized ranks of classifier guesses for each target emotion are depicted in Figure 2. Each point corresponds to the average normalized rank (from zero to one) of an emotion, for the target emotion in question. For example, in the classifier’s ranked output of possible identifications in *happiness* trials, shown in the uppermost line, *happiness* achieved the highest rank, 0.89, and *pride* achieved the second highest rank, 0.80. The nine target emotions in Figure 2 are ordered from top to bottom in terms of the rank accuracy of the correct response, from *happiness* to *envy*. For all emotions, the emotion ranked by the classifier as most likely was in fact the correct emotion.

Distances between emotions in Figure 2 correspond to relative differences between them according to the classifier on the basis of neural activation. Because the distances are relative, they depend on both the absolute difference between emotions as well as the set of emotions under study. Thus, for example, the relatively close correspondence between *happiness* and *pride* reflects both a similarity in their neural signatures and the fact that these were the only two positive emotions under study. In addition to this close correspondence of these two positive emotions, we found several noteworthy relationships: 1) anger was the negative emotion closest to the positive emotions, consistent with literature suggesting anger is positive in many respects [44] [45] [46]; 2) lust, a visceral state that some do not consider an emotion [47] [9], appears to be unlike both positive and negative emotions; 3) for negative emotions, the distance between an emotion and its nearest neighbor was greater for emotions considered “basic” (*disgust*, *fear*, *anger*, and *sadness*), than it was for non-basic emotions (*shame*, *envy*), suggesting that these emotions may in fact be more molar; and 4) several asymmetries exist. For instance, in *happiness* trials, *anger* achieved an average rank of 0.51, but in anger trials, happiness achieved an average ranking of just 0.33. This indicates that *anger* was more often confused with *happiness* when *happiness* was the target emotion than *happiness* was confused with *anger* when *anger* was the target emotion. More generally, the neural signatures for individual emotions provide an initial basis for relating emotions to one another.

**Figure 3:**
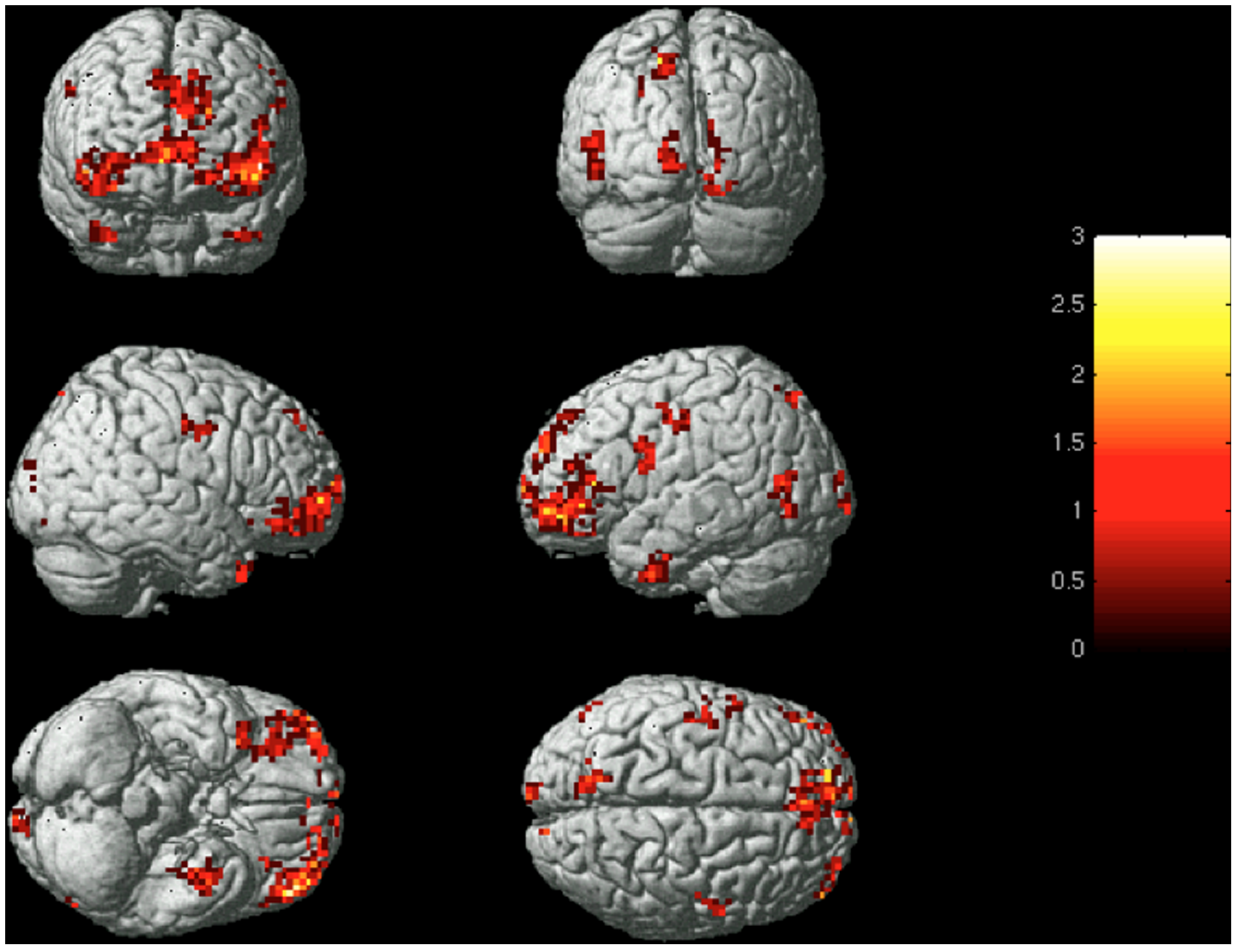
Group-level image of voxels used for within-subject classification. Images of the 240 most stable voxels across six presentations of emotion words were superimposed on one another, with resulting clusters of 25 or more voxels depicted. Color intensity reflects the number of participants for whom the voxel was among the 240 highest in stability.

Regions responsible for these classifications were distributed throughout the brain (see Figure 3). They include a large number of anterior frontal and orbital frontal voxels, a prevalence uncommon in neurosemantic classification studies of physical objects [40].

Notably, successful identification is not dependent on occipital activation that might capitalize on visual differences between the word prompts (i.e. activation which would allow for a classification based on word length, between the shortest (sad) and longest (embarrassed) words). When voxels in the occipital cortex were excluded from the classifier input, mean rank accuracy remained unchanged at 0.84 for within-subject classification, suggesting that the classification did not depend on a particular visual form. Though best classification accuracy was typically achieved with voxels taken from the entire brain, above chance classification rates could be achieved by selecting voxels from either the frontal, parietal, temporal, occipital, or subcortical regions in isolation (see Table 2), indicating that each of these regions encode emotion to a considerable degree. Thus, specific emotions can be decoded from distinct patterns of activation that are distributed across brain regions, but they can also be decoded from patterns of activation within a number of different individual brain regions. The distributed nature of neural activations associated with specific emotions provides further support to the results of recent meta-analyses which find that emotion inductions invoke a broad network of neural structures [14] [15].

**Table 2:** Classification Accuracies for Selected Brain Regions.

Region	Within Subject	Between Subject	Disgust Picture Classification
All	.84	.71	.91
All (Excluding Occipital)	.84	.70	.93
Frontal	.83	.66	.89
Parietal	.78	.67	.81
Temporal	.80	.73	.90
Occipital	.75	.68	.79
Subcortical	.76	.70	.76

### Factors Underlying Emotion-related Activation

To determine whether the activation patterns underlying the 18 emotions could be reduced to a small number of dimensions, we applied factor analysis to the activations of the stable voxels. The analysis revealed four factors that encode emotion-relevant concepts, as well as a fifth factor that encodes the length of the stimulus word. These five factors, explaining 40.1% of the variance in signal change, indicate properties of the emotions that drove neural responses (two additional factors were extracted but proved difficult to interpret; see Table 3). Each of these factors satisfied the K1 rule (i.e. eigenvalues greater than 1 [48]). They also meet the more conservative thresholds indicated by parallel analysis [49], generated through simulated data. As with any factor analysis, interpretation of factors is somewhat subjective. Below, we suggest plausible interpretations for these factors by examining converging information from three sources: 1) loadings of the 18 emotion words on the factors, 2) neural functions that have been consistently attributed to the locations of voxels underlying the factors, 3) significant correlations between factor scores and ratings of emotion along valence and arousal dimensions made by participants outside of the scanner, and 4) significant correlations between factor scores and ratings of the social nature of emotion made by a separate group of participants.

**Table 3:** Factor Scores for Emotion Words and Emotion Categories.

Factor	1	2	3	4	5	6	7
**Words**							
Angry	−0.56	−0.04	0.57	0.04	1.19	−0.57	−1.56
Enraged	−0.87	0.04	0.96	0.91	1.43	−0.81	−0.89
Disgusted	−0.47	2.24	−0.08	−1.29	−0.53	0.74	0.40
Revulsed	−0.73	1.83	0.08	−0.79	0.09	−0.59	0.54
Envious	−0.36	−1.36	0.88	−0.57	−0.19	1.19	−0.50
Jealous	−0.08	−1.75	0.91	−0.93	0.25	0.63	−0.73
Afraid	−0.50	0.46	−1.03	1.24	1.00	0.75	−0.20
Frightened	−0.88	−0.01	−1.18	1.75	0.74	2.05	0.68
Happy	1.55	0.32	−0.84	0.14	0.53	−1.14	−0.44
Joyous	1.81	0.76	−0.81	−0.18	0.32	0.48	−0.95
Horny	0.49	−0.64	−0.16	−1.92	2.09	−0.74	1.44
Lustful	0.53	−1.15	−0.53	0.57	−0.30	−0.36	2.69
Admirable	1.31	0.34	1.74	0.16	−0.88	0.69	0.64
Proud	1.97	−0.19	0.02	0.84	−1.08	0.28	−0.84
Gloomy	−1.07	−0.83	−1.62	−1.65	−1.52	0.79	−0.65
Sad	−0.62	−0.36	−1.31	0.32	−1.39	−2.11	−0.62
Ashamed	−0.90	−0.48	0.65	1.11	−0.87	−1.27	0.63
Embarrassed	−0.62	0.82	1.75	0.26	−0.87	−0.01	0.36
**Categories**							
Anger	−0.71	0.00	0.76	0.47	1.31	−0.69	−1.22
Disgust	−0.60	2.03	0.00	−1.04	−0.22	0.08	0.47
Envy	−0.22	−1.56	0.89	−0.75	0.03	0.91	−0.62
Fear	−0.69	0.22	−1.10	1.50	0.87	1.40	0.24
Happiness	1.68	0.54	−0.83	−0.02	0.42	−0.33	−0.69
Lust	0.51	−0.90	−0.35	−0.68	0.90	−0.55	2.06
Pride	1.64	0.08	0.88	0.50	−0.98	0.48	−0.10
Sadness	−0.84	−0.59	−1.46	−0.67	−1.46	−0.66	−0.63
Shame	−0.76	0.17	1.20	0.69	−0.87	−0.64	0.49
**Interpretation**	***Valence***	***Social***			***Arousal***	***Word Length***	***Lust***
% variance	9.13%	8.63%	7.85%	7.74%	7.62%	7.49%	7.26%

**Figure 4:**
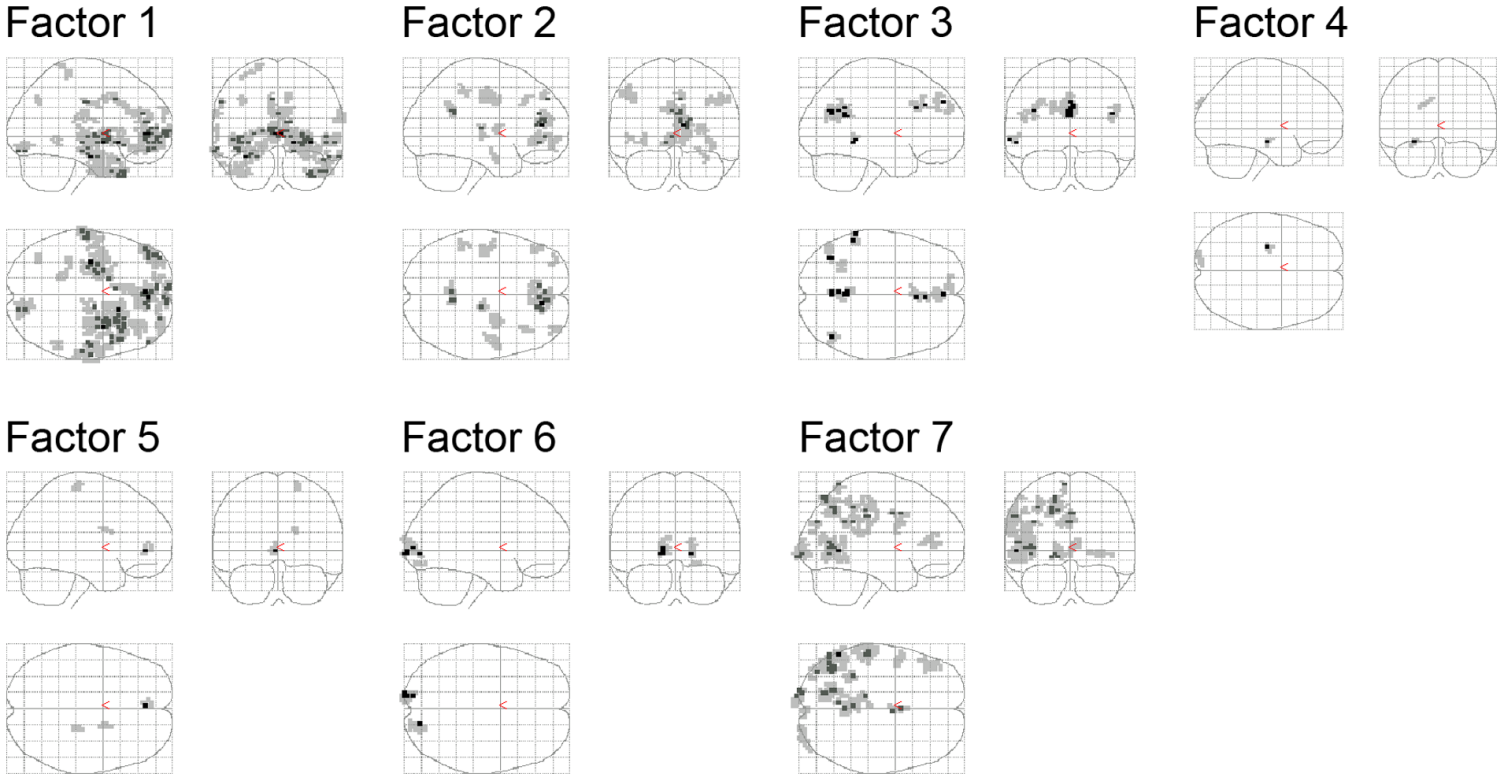
Voxel groups derived from factor analysis, threshold of 0.5, cluster size of 10. See text for additional details.

The factor explaining the greatest variance appeared to encode *valence*, the goodness or badness of the emotional situation. Positive words had positive *valence* factor scores, negative words had negatives scores, and lustful and horny (arguably ambiguous in their valence) fell in between (see Table 3). Factor scores correlated nearly perfectly *r*(18) = 0.96 (*p*<.001) with ratings of pleasantness of the emotional scenarios made by participants outside of the scanner (see Table 4). Neural regions underlying this factor were also consistent with a valence interpretation, including medial frontal regions implicated in core affect and emotion regulation [14] [50] [51] as well as orbital frontal and midbrain regions frequently associated with affective value computation [52] [53] [54] (see Figure 4 and Table 5).

**Table 4:** Factor Correlates.

	Pre-scan Ratings					Online Sample		
Factor	Pleasant	Arousing	Certain	Control	Attention	Valence	Person	Word Length
1	0.96	0.71	0.73	0.89	0.74	0.96	−0.17	−0.26
2	0.00	0.06	0.27	0.21	0.00	−0.10	0.55	0.35
3	−0.03	−0.05	0.12	0.01	−0.26	−0.01	−0.18	0.47
4	−0.08	0.12	−0.34	−0.23	0.16	−0.07	0.27	0.13
5	0.08	0.49	0.16	0.07	0.45	0.03	−0.26	−0.11
6	−0.02	−0.03	−0.22	−0.03	−0.06	0.03	0.37	0.55
7	0.15	0.27	0.05	0.11	−0.02	0.20	−0.13	0.38

Correlations between factor scores and ratings made by participants prior to scanning session, ratings made by independent online sample, and length of stimulus words.

**Table 5:** Neural Clusters Identified by Factor Analysis.

Factor	Label	x	y	z	No. of Voxels	Radius (mm)
1	Frontal_Sup_Medial_R	6	52	5	187	16
	Putamen_R	25	5	−3	105	12
	Frontal_Mid_Orb_L	−41	49	−6	88	9
	Temporal_Pole_Mid_R	41	14	−35	84	9
	Hippocampus_L	−28	−13	−19	47	9
	Cerebellum	20	−47	−23	38	10
2	Cingulum_Ant_R	9	46	18	44	8
	Postcentral_L	−46	−13	43	43	7
	Cingulum_Post_L	0	−50	29	38	8
	Precentral_R	49	−7	38	32	6
	Cingulum_Ant_L	−7	37	−5	24	6
	Putamen_R	28	−3	8	23	5
3	Frontal_Sup_Medial_L	0	35	35	54	12
	Precuneus_L	−1	−56	26	42	7
	Occipital_Mid_L	−43	−68	24	29	7
	Frontal_Sup_L	−12	57	29	28	6
	Angular_R	45	−64	25	23	6
	Temporal_Mid_L	−58	−42	−2	16	4
4	Occipital_Sup_L	−15	−94	31	15	5
	Hippocampus_L	−27	−14	−15	10	4
5	Caudate_R	18	3	22	11	4
	Precentral_R	20	−27	67	11	4
	Cingulum_Ant_L	−5	46	2	10	5
6	Occipital_Sup_L	−12	−96	5	39	7
	Calcarine_R	17	−89	2	22	6
	Lingual_R	21	−84	−14	13	4
7	Temporal_Mid_L	−49	−65	3	186	12
	SupraMarginal_L	−59	−30	33	80	13
	Parietal_Sup_L	−17	−67	47	72	9
	Cingulum_Post_L	−3	−48	31	45	8
	Calcarine_L	−12	−100	−2	37	6
	Frontal_Inf_Tri_L	−45	37	10	36	8

Threshold of 0.5, minimum cluster size of 10. For each factor, the six largest clusters are shown.

A second factor appeared to correlate with *arousal* or preparation for action. Factor scores correlated with subjective ratings of arousal (*r*(18) = 0.49, *p* = .04; see Table 4). Anger, fear and lust categories had the highest scores, whereas sadness, shame and pride had the lowest. Few clusters of voxels were identified as underlying this factor, but those that were support an arousal interpretation. These include areas of the Basal Ganglia and Precentral Gyrus (see Figure 4 and Table 5) implicated in action preparation, and a medial frontal region both anatomically and functionally connected to periaqueductal gray and the hypothalamus, regions thought to regulate physiological response to affective inductions [13].

A third factor encoded whether or not the emotion had a *social* element (i.e., another person [35]). Physical disgust and revulsion, least likely to involve another person, had the highest scores, whereas jealousy, envy, horny, and lustful, all requiring a specific other, had the lowest scores. To test this interpretation, we asked an online sample of 60 participants to come up with scenarios for each emotion word, and then asked whether or not their scenario involved another individual. Scores of the *social* factor correlated significantly with the presence of another person (*r*(18) = 0.55, *p* = .02, see Table 4). Voxels underlying the *social* factor came primarily from anterior and posterior areas of the cingulate cortex. These ‘default network’ regions have frequently implicated in person perception [55].

A fourth factor appeared to uniquely identify *lust*, separating it from other emotion categories. Lustful and horny had the highest scores, and no other emotion category had high factor scores (see Table 3). A separate factor identifying lust is consistent with classification results that suggest that no other emotion category was frequently confused with it (see Figure 2). Neural regions associated with this factor include the fusiform gyrus and inferior frontal areas implicated in face processing [25] [56], as well as areas that overlap substantially with those previously reported in the processing of sexual stimuli [57].

Finally, one factor encoded *word length*. Factor scores correlated with length of stimulus words (*r*(18) = 0.55, *p* = .02; see Table 4), with *frightened* showing the highest score, and *sad* the lowest. Neural regions underlying this factor showed clusters only in the occipital cortex (note that successful classification did not depend on occipital cortex, see Table 2).

## Discussion

We used a Gaussian Naïve Bayes pooled variance classifier to show that specific emotional states can be identified on the basis of their neural signatures, and that these signatures are reliably activated across episodes and across individuals. For the only emotion category tested (disgust), the results further indicate reliable activation across different types of emotional experience. Factor analysis suggests that groups of voxels associated with valence, arousal, sociality and lust underlie the successful classifications. The results inform our understanding of emotional processes and suggest the potential to infer a person’s emotional reaction to stimuli on the basis of neural activation.

The results may also shed some light on a contentious debate in the psychology of emotion. Acceptance of the biologically basic view of emotions has been hampered by the failure to identify neural signatures associated with specific emotions. The reliability of classifications achieved suggests that such signatures do exist and that they share commonality across individuals. Classification accuracies achieved thus suggest the possibility of a neural architecture for emotion, and in so doing provide modest support for a biologically basic view.

However, the results are not inconsistent with constructionist theories – cognitive constructions could likewise display specific and identifiable patterns. Moreover, the factors found to underlie neural activations include valence and arousal, which feature prominently in constructionist theories [58] [59] [60]. They emerge here from a data-driven analysis and their specification includes a set of associated neural regions. Other factors are less frequently included as building blocks of emotion, such as a social factor suggesting that the neural representation of certain emotions includes representation of interpersonal interaction, and a lust factor that is distinct from general arousal. Despite its prominence in the literature, we did not find a factor corresponding to approach/avoidance. These patterns in the neural activation underlying emotions may provide insight into their psychological organization.

Also consistent with the constructionist view, each of the factors identified encompasses voxels in regions linked to wide variety of functions, including primary sensory and motor functions, cognitive conflict, person and self-perception, and top-down processes like emotion regulation. Valence, arousal, lust, and social presence are not represented in single brain regions. This diversity speaks to the fact that emotional experiences, like most other complex thoughts, are represented in a broad array of neural circuits.

In sum, the results point to a middle ground in the basic vs. constructionist debate. Decomposable and identifiable patterns of activation characteristic of specific emotions exist and vary in predictable ways: the neural signature of anger is different than that of sadness, which is different than that of disgust. These signatures share commonality across emotional episodes, across individuals and across different modes of emotion induction. But these patterns are not solely comprised of dedicated emotional circuitry. Though they are distinguishable, they are far from simple, and what best differentiates emotions at the neural level may include concepts not typically thought of as emotional [61] [13] [58].

Beyond their theoretical implications, the present results suggest a method of measuring emotional response which can complement existing techniques. In general, development of reliable measures of specific emotion has proven difficult. Self-report, still the gold standard [62], is vulnerable to deception and demand effects and has severe limitations when one accepts the view that some emotions are not experienced consciously [63] [64]. Physiological measures such as heart rate and skin conductance show some ability to discriminate between broad categories of emotion but have limited ability to make finer classifications [65] [66]. Facial expressions have also been used to categorize a subset of emotions [67], but emotions can occur in the absence of facial expressions and facial expressions can occur in the absence of emotion [68]. Neural circuits that mediate certain emotion-related behaviors (e.g. freezing, [69] [70]) have been identified, but researchers have yet to achieve reliable identification of emotions on the basis of neural activation [8]. In short, existing methods of emotion measurement suffer from a variety of limitations [71].

The present experiment provides the first steps towards a novel technique for emotion identification. Specific emotions were identified on the basis of neural activation reliably, even when classifier training used separate subjects. Moreover, a classifier trained on imagined emotional experiences reliably identified the emotional content of stimuli from an entirely different modality. The systematicity of the neural activation patterns suggests the possibility of producing a generative model that could predict an individual’s emotional response to an arbitrary stimulus (e.g. a flag, a brand name, or a political candidate).
